# *Auricularia auricula* Anionic Polysaccharide Nanoparticles for Gastrointestinal Delivery of *Pinus koraiensis* Polyphenol Used in Bone Protection under Weightlessness

**DOI:** 10.3390/molecules29010245

**Published:** 2024-01-02

**Authors:** Li Kang, Qiao Li, Yonghui Jing, Feiyan Ren, Erzhuo Li, Xiangyin Zeng, Yier Xu, Dongwei Wang, Qiang Wang, Guicai Sun, Lijun Wei, Yan Diao

**Affiliations:** 1 College of Life Science, China West Normal University, Nanchong 637009, China19108175785@163.com (Y.J.); rfy030928@163.com (F.R.);; 2 School of Life Science and Technology, Harbin Institute of Technology, Harbin 150000, Chinas1005674406@163.com (E.L.);; 3 The First Affiliated Hospital of Nanchang University, Nanchang 330000, China; 4 Collaboration Innovation Center for Tissue Repair Material Engineering Technology, China West Normal University, Nanchong 637002, China

**Keywords:** *Auricularia auricula* anionic polysaccharides, nanoparticles, self-assembly, *Pinus koraiensis* polyphenol, food additives

## Abstract

*Auricularia auricula* polysaccharides used in *Pinus koraiensis* polyphenol encapsulation and delivery under weightlessness are rarely reported. In this study, an anionic polysaccharide fragment named AAP Iα with a molecular weight of 133.304 kDa was isolated and purified to construct a polyphenol encapsulation system. Nanoparticles named NPs-PP loaded with a rough surface for *Pinus koraiensis* polyphenol (PP) delivery were fabricated by AAP Iα and ε-poly-L-lysine (ε-PL). SEM and the DLS tracking method were used to observe continuous changes in AAP Iα, ε-PL and PP on the nanoparticles’ rough surface assembly, as well as the dispersion and stability. Hydrophilic, monodisperse and highly negative charged nanoparticles can be formed at AAP Iα 0.8 mg/mL, ε-PL 20 μg/mL and PP 80 μg/mL. FT-IR was used to determine their electrostatic interactions. Release kinetic studies showed that nanoparticles had an ideal gastrointestinal delivery effect. NPs-PP loaded were assembled through electrostatic interactions between polyelectrolytes after hydrogen bonding formation in PP-AAP Iα and PP-ε-PL, respectively. Colon adhesion properties and PP delivery in vivo of nanoparticles showed that NPs-PP loaded had high adhesion efficiency to the colonic mucosa under simulated microgravity and could enhance PP bioavailability. These results suggest that AAP Iα can be used in PP encapsulation and delivery under microgravity in astronaut food additives.

## 1. Introduction

Astronauts suffer from bone loss during long-term space missions, which is difficult to recover from after returning to the ground [1]. Previous studies have shown that *Pinus koraiensis* polyphenols (PP), which are mixtures containing multiple phenolic compounds, have biological functions against microgravity-induced bone loss [2]. The chemical structure of PP is unstable [3] because it can be easily oxidized and lose its biological activity during food processing in space missions. In addition, the biological activity of PP can be reduced or completely lost through the gastrointestinal tract [4]. Moreover, physiological changes under microgravity that cause poor contact between gastrointestinal contents and tract, as well as the increased gastrointestinal peristalsis and shortened gastrointestinal emptying time, can be greatly impeded by PP absorption [5]. These factors make it almost impossible for PP to be applied to aerospace food in practical space missions.

The use of polyelectrolyte complexes (PECs) to encapsulate polyphenols is a popular trend in solving the application problems of polyphenolic compounds due to their unstable structure, poor water solubility and unpleasant odor. PECs are formed by the electrostatic interaction between two or more polyelectrolytes with opposite charges [6]. In the current, there are various materials and methods for PEC forming; however, the systems that assemble PECs generally include polysaccharide-peptide and polysaccharide-polysaccharide systems. Around these methods, materials and systems, PECs with a polysaccharide surface exhibit good mucoadhesive properties [7].

Edible mushroom polysaccharides are a type of natural macromolecule composed of more than 10 monosaccharides linked by glycosidic bonds. They can be isolated from the fruiting body and composed of heteropolysaccharides. The heteropolysaccharide composition structure can be prepared into PECs with various properties [8,9,10,11]. In recent years, it has become a trend to develop polysaccharides from edible fungi as encapsulation materials. *Auricularia auricula* polysaccharides (AAP) are heteropolysaccharides that mainly consist of glucans, mannose, galactose or multiple glycuronate [12,13]. Most of the research on AAP has focused on their biological effects (e.g., antioxidant activity [14], antitumor [15], hypolipidemic activity [16], etc.). However, there are few reports on the utilization of AAP with anion in nanodrug delivery systems.

ε-poly-L-lysine (ε-PL) is a cationic polymer, consisting of 25 to 35 lysine residues, which has antibacterial activity. It has been reported that ε-PL has excellent water solubility, thermal stability and safety. ε-PL can be broken down into lysine, an essential amino acid for the human body [17], which is then absorbed and used without accumulation in the body [18]. The use of ε-PL is common in food applications, such as cooking rice, cooked vegetables, soup, noodles and sliced fish (sushi).

The aim of this study is to develop an astronaut food additive by using anion AAP. Compared with other medical polysaccharide materials, AAP is of food grade, without considerable side effects. Astronaut food additives could be served as gastrointestinal delivery systems to enhance polyphenolic compound absorption and bioavailability under microgravity. In the present study, a series of PECs were constructed using *Auricularia auricula* anionic polysaccharide fragments and ε-PL, because there are few reports on the encapsulation of polyphenol mixtures using anionic AAP. Then, the encapsulation efficiencies of PECs on PP were screened. The developed PECs were characterized using SEM, DLS and FT-IR. The formulations were optimized and the release curve of PP in simulated human gastrointestinal fluid was obtained. Finally, hindlimb-unloaded rats were used as a simulated microgravity animal model to evaluate the colonic adhesion properties and deliver efficiencies of the PECs.

## 2. Results

### 2.1. Extraction, Purification and Molecular Weight of the Anionic Polysaccharide from Auricularia auricular

**Figure 1:**
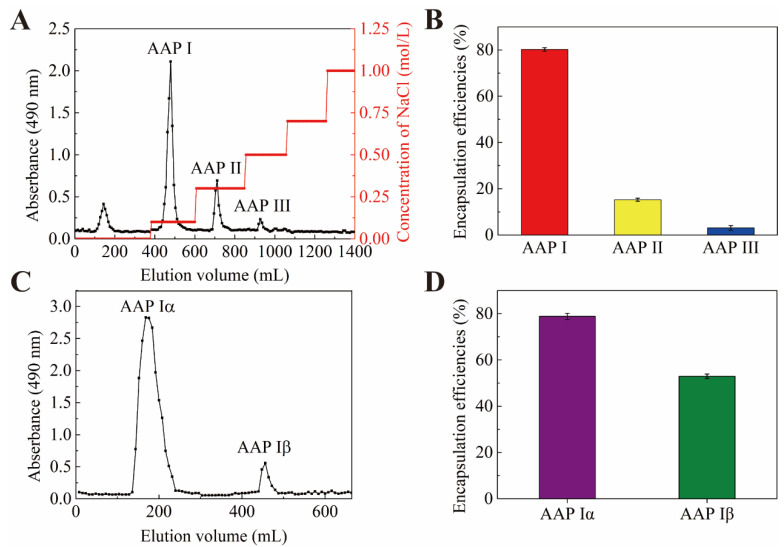
Elution curve of AAP on DEAE-52 (**A**). Encapsulation efficiencies of PP (**B**,**D**). Elution curve of AAP I on Sephadex G-100 gel chromatography column (**C**).

AAP was eluted with a DEAE-52-cellulose column and four fractions were obtained (Figure 1A). One fraction was eluted by distilled water and the others (named AAP I, AAP II and AAP III) were eluted by 0.1, 0.3, 0.5 M of NaCl, respectively. In order to figure out which fraction has the potential to encapsulate PP, the encapsulation efficiencies of the NPs-PP loaded were assessed. The findings suggested that AAP I had the highest encapsulation efficiency among the anionic polysaccharides from *Auricularia auricular* (Figure 1B). Therefore, AAP I was purified through sephadex-G100 and two fractions named AAP Iα and AAP Iβ were obtained, respectively (Figure 1C). AAP Iα exhibited higher PP encapsulation efficiencies (Figure 1D). These data indicate that AAP Iα is the most suitable anionic polymer for encapsulating PP among all anionic polysaccharides. Therefore, AAP Iα was used in the continued research on PP encapsulation.

The molecular weight of AAP Iα was determined by GPC. It can be seen that Mn of AAP Iα was 110,144 Da, Mw was 133,304 Da (Figure 1A), Mp was 145,663 Da, and Mw/Mn was 1.210 (Table 1).

### 2.2. SEM Tracking of Rough Surface Assembly of NPs-PP Loaded 

**Figure 2:**
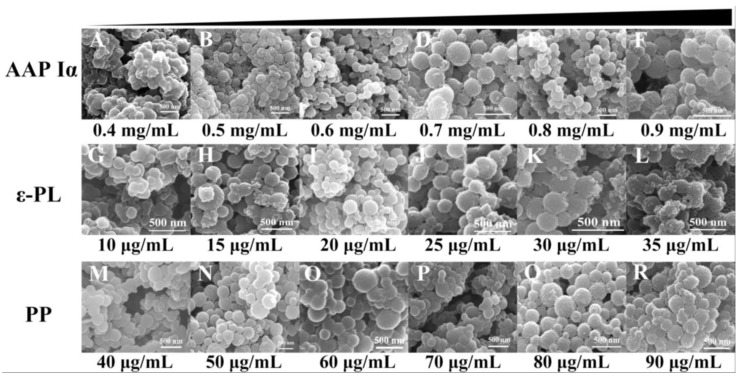
Morphology of NPs-PP loaded under SEM. SEM photographs of the morphology of AAP Iα (**A**–**F**), ε-PL (**G**–**L**) and PP (**M**–**R**) at different concentrations of NPs-PP loaded, respectively.

To construct NPs-PP loaded with rough surfaces, the nanoparticle formulations were investigated and the SEM tracking method was employed (Figure 2). The results showed that NPs-PP loaded had severe adhesion and a spherical surface could not be formed at low (0.4 mg/mL) or high (0.9 mg/mL) concentrations of AAP Iα in the assembly system (Figure 2A,F). As AAP Iα concentration gradually increased, a rough surface was exhibited when NPs-PP loaded began to form a spherical surface (Figure 2B–E). This rough surface was not affected by AAP Iα concentration changes. ε-PL had a significant impact on NPs-PP loaded spheroidization. Well-formed NPs-PP loaded could be obtained only at ε-PL of 20–25 μg/mL (Figure 2I,J). SEM morphology of nanoparticles changed slightly with the increase in PP concentration (Figure 2M–R). The above results indicated that NPs-PP loaded with rough surfaces could be constructed by adjusting concentrations of AAP Iα, ε-PL and PP in the assembly system. Additionally, AAP Iα and ε-PL have a significant effect on the morphology of nanoparticles.

### 2.3. DLS Tracking Size Distributions of NPs-PP Loaded 

**Figure 3:**
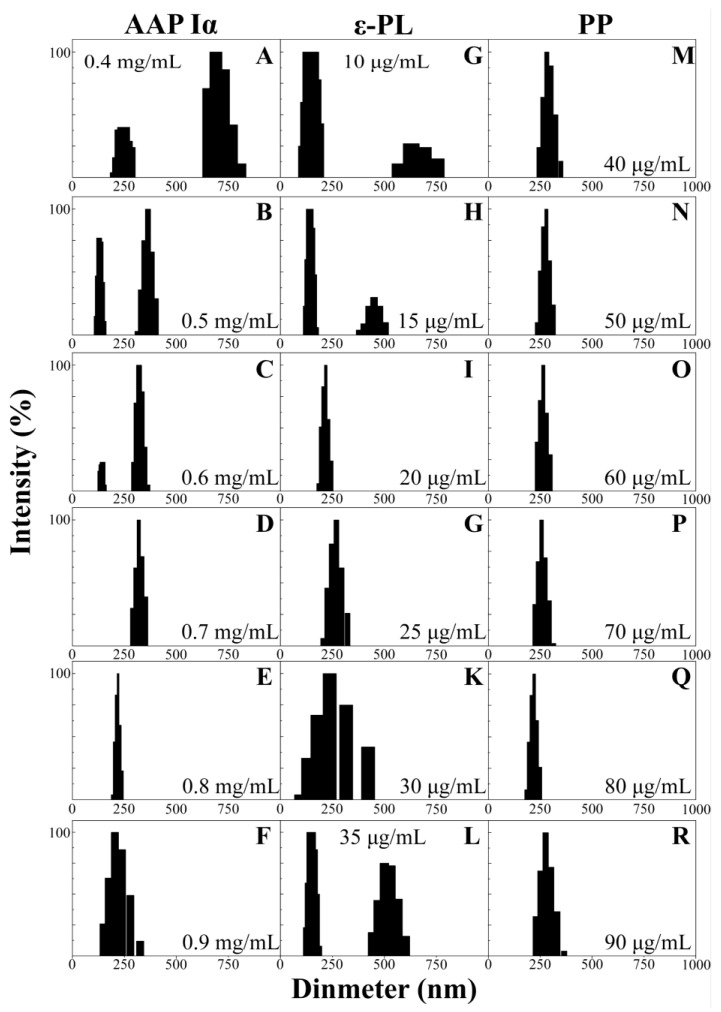
DLS data of NPs-PP loaded size distributions. Effects of different concentrations of AAP Iα (**A**–**F**), ε-PL (**G**–**L**) and PP (**M**–**R**) on NPs-PP loaded size distributions.

The size distributions exhibited a trend which narrowed first and then widened following AAP Iα, ε-PL and PP concentration changes (Figure 3A–R). The distribution range of NPs-PP loaded gradually decreased with increasing AAP Iα concentration in the assembly system. The split peak disappeared at AAP Iα of 0.7 mg/mL in the size distribution diagram (Figure 3D). As AAP Iα concentration increased in the assembly system, the figures showed that the distribution peak narrowed first and then widened. The narrowest nanoparticles size distribution range was presented at AAP Iα of 0.8 mg/mL, from 192 to 240 nm (Figure 3E). The effects of ε-PL on the range form of NPs-PP loaded exhibited a decrease first and then an increase, similar to the trend of AAP Iα (Figure 3G–L). At ε-PL of 20 μg/mL, the split peak disappeared, and the narrowest peak range was exhibited between 180 to 247 nm (Figure 3I). Compared with AAP Iα and ε-PL, the effect of PP in the assembly system is relatively weak. When PP is 80 μg/mL, the narrowest range of NPs-PP loaded was reached between 182 and 251 nm (Figure 3Q). These results suggested that dispersion and homogeneity of NPs-PP loaded was profoundly impacted by concentrations of AAP Iα and ε-PL, while PP had little effect on nanoparticle size distribution.

### 2.4. PP Loading and Stability

**Figure 4:**
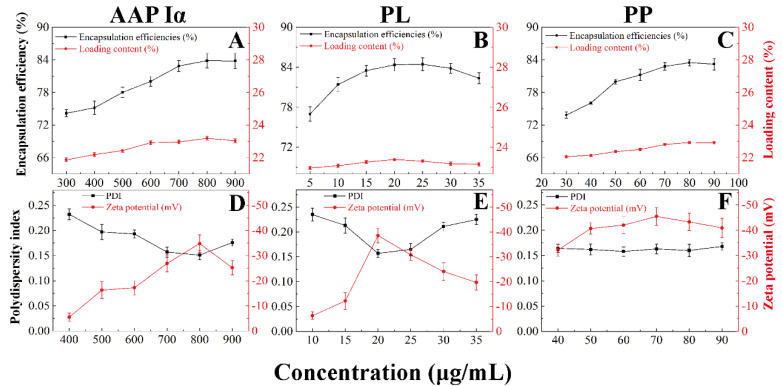
Effects of different concentrations of AAP Iα, ε-PL and PP on encapsulation efficiencies and loading content (**A**–**C**), and polydispersity index and zeta potential (**D**–**F**) of NPs-PP loaded, respectively. Each point represents the mean value ± standard deviation (n = 3).

To further research the encapsulation effect of PECs formed by AAP Iα and ε-PL on PP and the stability of NPs-PP loaded, the encapsulation efficiencies, loading contents, PDI and zeta potential of the nanoparticles were tested (Figure 4A–F). With the change in AAP I α, ε-PL and PP concentration, the entrapment efficiency and drug loading of NPs-PP loaded increased at first and then decreased (Figure 4A–C). However, the opposite trend was presented in PDI and zeta potential figures (Figure 4D–F and Table 2). Among these data, the most significant impact factor was ε-PL. Encapsulation efficiencies and loading contents reached the highest value at AAP Iα of 0.8 mg/mL, ε-PL of 20 μg/mL and PP of 80 μg/mL, while the lowest point appeared in the PDI and zeta potential diagram, showing the best stability of the self-assembly system simultaneously. These data implied that AAP Iα and ε-PL had a significant impact on the stability of NPs-PP loaded, which is consistent with the result of the morphology and size distribution observations. In summary, the optimized formulation for NPs-PP loaded is AAP Iα of 0.8 mg/mL, ε-PL of 20 μg/mL and PP of 80 μg/mL, and these were used in subsequent studies.

### 2.5. FT-IR

**Figure 5:**
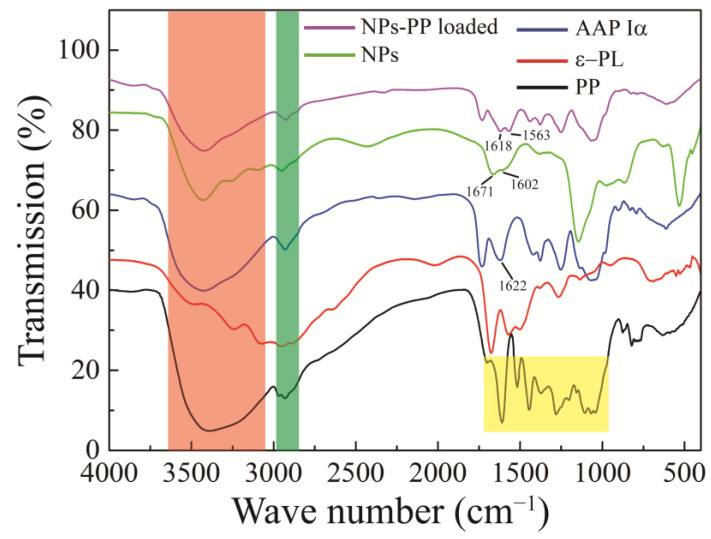
FT-IR spectra of PP, ε-PL, AAP Iα, NPs and NPs-PP loaded. The AAP Iα, PP, NPs, and NPs-PP loaded spectra all display broad absorption peaks ranging from 3000 to 3600 cm^−1^ (indicated by red shading) and weaker absorption peaks between 2900 and 3000 cm^−1^ (marked by green shading). Additionally, the PP spectrum reveals a series of absorption peaks in the 1700–1000 cm^−1^ range (highlighted in yellow).

FT-IR was used to analyze the intermolecular interactions. FT-IR of PP, ε-PL, AAP Iα, NPs and NPs-PP loaded are illustrated in Figure 5. There were wide absorption peaks distributed from 3000 to 3600 cm^−1^ (red color shadow) and weak absorption peaks at 2900~3000 cm^−1^ (green color shadow) in the AAP Iα, PP, NPs and NPs-PP loaded spectra. The FT-IR spectrum of AAP Iα showed one sharp peak and one weak peak at 1622 cm^−1^ and 1420 cm^−1^, respectively. Three absorption peaks appeared in the ε-PL curve which represent amide I (1673 cm^−1^), amide II (1562 cm^−1^) and amide III (1256 cm^−1^), respectively. A series of absorption peaks in the range of 1700–1000 cm^−1^ (yellow color shadow) were observed in the PP spectrum. Compared with PP, polyphenol characteristic peaks on the NPs-PP loaded FT-IR spectrum disappeared. Additionally, compared with AAP Iα, a strong peak at 1622 cm^−1^ was divided into two peaks at 1671 and 1602 cm^−1^ on the NPs spectrum and 1618 and 1563 cm^−1^ on the NPs-PP loaded spectrum, respectively.

### 2.6. Release Kinetics

**Figure 6:**
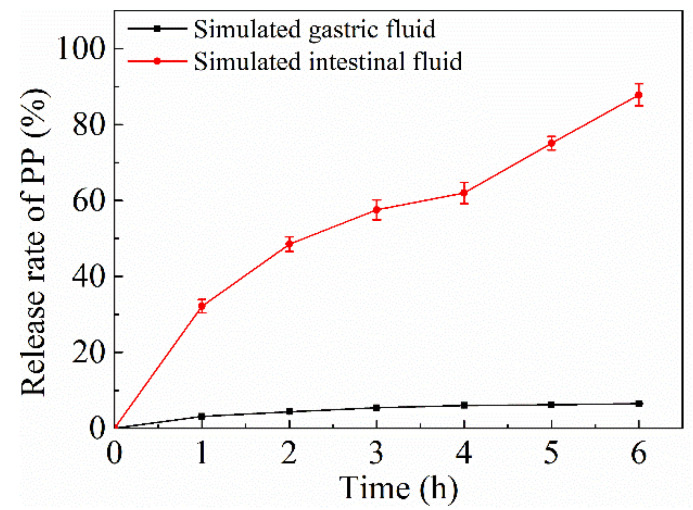
In vitro release of PP from NPs-PP loaded in SGF and SIF. Each point represents the mean value ± standard deviation (n = 3).

The NPs-PP loaded release profile in SGF and SIF was studied (Figure 6). A low release rate of PP (8.5 ± 0.14%) was observed after incubation for 6 h in SGF. However, there is one completely different PP release trend in SIF. Compared with SGF, PP released rapidly in SIF. The release rate was relatively gentle in the initial 4 h, indicating that NPs-PP loaded had a gradual release process in SIF. After 4 h, the PP release process suddenly intensified, and the release rate reached about 90% after incubation for 6 h. The characteristic that PP released slowly in the stomach but fast in the intestines presupposes that NPs-PP loaded are an ideal oral carrier. Mathematical models were constructed for the PP release situation in different dissolution media (Table 3). It can be concluded from the results that the release profiles of PP in SGF and SIF both showed higher R^2^ for the Ritger-Peppas model.

### 2.7. Adhesion Properties of NPs-PP Loaded on Colon Ex Vivo

**Figure 7:**
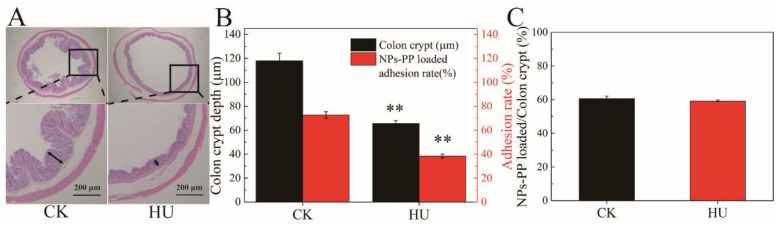
Adhesion effect of NPs-PP loaded on colon ex vivo. H&E staining of the colon (**A**). Image J analysis data for H&E staining and adhesion rate of NPs-PP loaded on colon (**B**). Analysis data of NPs-PP loaded/colon crypt between CK and HU (**C**). The statistical results shown represent the mean ± SD (n = 3), boxes represent enlarged tissue parts, arrow indicates colon crypt depth, vs. the CK group, ** *p* < 0.01. n = 6 rats for each group. CK: ground group. HU: hindlimb-unloaded group.

The HU group were used as simulated microgravity models to study colon morphology and adhesion properties of NPs-PP loaded. Intact colon tissue structure, smooth mucosa, clear layers and an undamaged muscle layer were detected in the CK group. However, the number of crypts on the colon were decreased, colon folds were reduced and the lamina propria was loose in the HU group (indicated by the black arrow) (Figure 7A). Image J software was used to quantitatively represent the destructive effect of simulated microgravity on the colon. The results showed that crypts number in the HU group was significantly decreased compared with the CK group (Figure 7B). The adhesion rate of NPs-PP loaded was also significantly decreased. However, there was no significant difference in NPs-PP loaded/colon crypt% data between the CK group and the HU group (Figure 7C), indicating that the adhesion properties of NPs-PP loaded did not change owing to its rough surface structure. These results manifested that colon tissue structure was destructed by microgravity, and NPs-PP loaded had good adhesion properties on the colon.

### 2.8. Delivery Effects of NPs-PP Loaded on PP under Simulated Microgravity In Vivo

**Figure 8:**
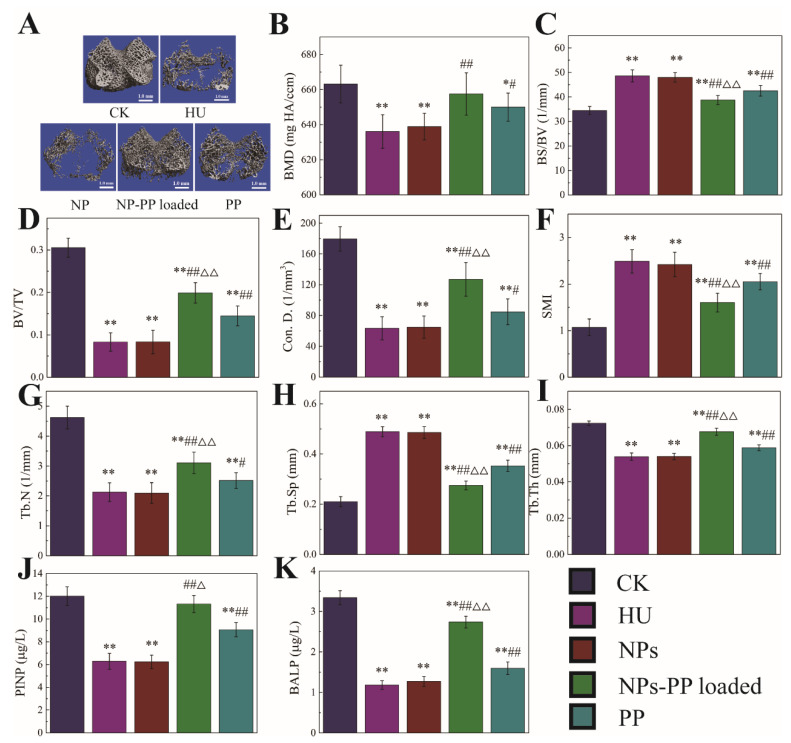
Micro-CT analysis of the cancellous area from the distal femur and PINP and BALP concentration in serum under simulated microgravity. Cancellous: one 2.1 mm thick trabecular bone chip under the epiphyseal plate in the lower end of the femur was selected as the region of interest (ROI); scale: 1 mm. 3D reconstruction images of cancellous ROI in femur (**A**). BMD (**B**), bone mineral density (mg HA/ccm), refers to the total BMD of the ROI. BS/BV (**C**), bone surface to bone volume (1/mm), refers the content of bone tissue in the sample. BV/TV (**D**), bone volume fraction, refers to the ratio of bone volume to tissue volume. Con.D (**E**), connectivity density, shows the number of connections in the trabecular networks. SMI (**F**), structure model index, is a method for determining the plate- or rod-like geometry of trabecular structures. Tb.N (**G**), trabecular number (1/mm). Tb.Sp (**H**), trabecular separation (mm). Tb.T (**I**), trabecular thickness (mm). PINP (**J**) and BALP (**K**) concentration in serum. The statistical results shown represent the mean ± SD. vs. the control group, * *p* < 0.05, ** *p* < 0.01, vs. the HU group, # *p* < 0.05, ## *p* < 0.01, vs. the PP group, △ *p* < 0.05, △△ *p* < 0.01. n = 6 rats for each group. CK: ground group. HU: hindlimb-unloaded group. NP: rats were treated with 118.75 mg/kg/d nanoparticles without PP loaded during hindlimb-unloading. NP-PP loaded: rats were treated with 156.25 mg/kg/d nanoparticles with PP loaded during hindlimb-unloading. PP: rats were treated with 37.5 mg/kg/d polyphenols isolated from pine (*Pinus koraiensis*) during hindlimb-unloading.

In order to validate the delivery effect in vivo, the mechanical properties of rat femurs (Figure S2A–E), the structure of the cancellous area from the distal femur (CADF) (Figure 8A–I), and PINP and BALP in the serum (Figure 8J,K) were studied. The mechanical properties of femurs found that decreased mechanical properties caused by HU were significantly alleviated by NPs-PP loaded. However, there were no significant differences between the NPs-PP loaded group and the PP group, although the trend of data in the NPs-PP loaded group was better than that in the PP group. Contours and order of tissue of the CADF in the NPs-PP loaded group was better than that in the PP group (Figure 8A). There was no significant difference in BMD between the NPs-PP loaded group and the PP group. However, the data for the BS/BV, BV/TV, Con. D., SMI, Tb.N., Tb.Th. and Tb.Sp. of the CADF in the NPs-PP loaded group were significantly higher than those in the PP group (Figure 8C–I). The levels of PINP and BALP in the NPs-PP loaded group were significantly higher than those in the PP group. These results indicated that NPs-PP loaded could significantly alleviate bone loss caused by HU in rats and with better results than PP alone. All of these data suggested that PP bioavailability was significantly improved by AAP Iα encapsulation.

## 3. Materials and Methods

### 3.1. Materials and Regents

Polyphenols of *Pinus koraiensis* (PP) were given by Professor Zhenyu Wang at the Harbin Institute of Technology. *Auricularia auricula* were purchased from Daxing’anling, China. Conductive tape was purchased from Zhongjing Science and Technology Co., Ltd. (Beijing, China). ε-Poly-L-lysine (ε-PL, MW: 150,000–300,000) was purchased from Solarbio (Beijing, China). Other analytical reagents were purchased from Sigma Aldrich (Shanghai, China) Trading Co., Inc. Sprague-Dawley rats were purchased from the Animal Center of Harbin Medical University (Harbin, China).

### 3.2. Extraction and Purification of Anionic Polysaccharide Fragments from Auricularia auricula

The dried *Auricularia auricula* was crushed into powder and sifted through a 60-mesh sieve. Crude polysaccharides of *Auricularia auricula* (AAP) were extracted following the method of Liu et al. [19]. *Auricularia auricula* powder was mixed with distilled water (1:100, *w*/*v*) at 80 °C for 4 h. During this period, ultrasound-assisted extraction (80 °C, 500 W) was performed 3 times for 30 min each time. The supernatants were collected after centrifugation (4000 rpm, 20 min) and then mixed with hydrogen peroxide at 37 °C for 24 h to remove pigments. After being dialyzed with distilled water for 24 h, the solution in the dialysis bag was mixed with Sevag reagent (chloroform/n-butanol 3:1, *v*/*v*) at a ratio of 5:1 (*v*/*v*) under the condition of stirring for 4 h. The aqueous phase was collected and added with ethanol (1:3, *v*/*v*), and then, the mixture was kept at 4 °C for 48 h. AAP was obtained by centrifugation (4000 rpm for 20 min, 4 °C) and vacuum freeze-drying.

AAP I, AAP II and AAP III was obtained by anion-exchange methods [20]. Crude polysaccharides of *Auricularia auricula* were prepared into 2 mg (Glucose equivalent)/mL solution and purified by anion-exchange chromatography (DEAE-52-cellulose chromatography column, 2.5 cm i.d. × 40 cm). After sample loading (1/10 column volume), distilled water, 0.1 M NaCl, 0.3 M NaCl and 0.5 M NaCl was eluted at 0.4 mL/min flow rate. After being dialyzed with distilled water for 24 h, AAP I, AAP II and AAP III were obtained from 0.1, 0.3 and 0.5 M NaCl eluent, respectively.

Isolation and purification of AAP Iα and AAP Iβ was achieved by molecular exclusion methods [21]. AAP I, which was further purified by molecular exclusion chromatography (Sephadex G-100 chromatography column, 2.5 cm i.d. × 40 cm), was prepared into 10 mg (Glucose equivalent)/mL. After sample loading (1/10 column volume), distilled water was eluted at 0.2 mL/min. AAP Iα and AAP Iβ were collected, respectively.

Polysaccharides were identified by the phenol-sulfuric acid method at 490 nm [22]. Samples were mixed 1 mL of 5% phenol and 5 mL of sulfuric acid in water bath at 100 °C for 20 min, respectively. Distilled water was used instead of the sample as a blank control. After cooling, the sample mixture was scanned at 490 nm.

### 3.3. Molecular Weight Analysis

The molecular weight analysis referred to Ponnudurai and Zinovyev et al. for the experimental methods [23,24]. TDA305max multi detector gel permeation chromatography (GPC) produced in the UK (Malvern Panalytical, Great Malvern) was used to determine the weight average molar mass (Mw) and the number average molar mass (Mn) of the polysaccharides. Samples of 100 μL were eluted with 0.1 mol/L NaNO_3_ and filtered via a 0.22 μm PVDF membrane. The flow rate was 0.7 mL/min (45 °C). TDA305max was conducted by an acquisition operator and calculation operator. Moreover, polyethylene glycol was used as standard for data analysis.

### 3.4. Preparation of NPs-PP Loaded

NPs-PP loaded were constructed by polyelectrolyte self-assembly technology [25]. AAP Iα, ε-PL and PP were mixed in a tube (5 mL, pH 8.0) with different concentrations, shown in Table 4, respectively, and then, the tube was put on a thermostatic culture oscillator (ZHWY-211C, Shanghai Zhicheng Analytical Instrument Co., Ltd., Shanghai, China) at 30 °C with 100 rpm. Three hours later, the NPs-PP loaded were obtained by centrifugation (8000 rpm for 5 min) at 20 °C.

PP was determined by the modified Folin-Ciocalteu method [26]. The samples were mixed with 1 mol/L Folin-Ciocalteu reagent at a ratio of 1:1, respectively. Five minutes later, Na_2_CO_3_ solution (5%, *w*/*v*) was added into the sample mixture (Na_2_CO_3_ solution/sample mixture 2:1, *v*/*v*). The absorption value (760 nm) of the mixtures was measured by a microplate reader (infiniteM200, TECAN, Morgan Hill, CA, USA), while distilled water was used instead of the sample as a blank control.

The encapsulation efficiency (*EE*) and loading content (*LC*) of the NPs-PP loaded were calculated using the following equations:

*EE* (%) = (*P_T_* − *P_S_*)*/P_T_* × 100%

*LC* (%) = (*P_T_* − *P_S_*)*/M* × 100%

*EE*: encapsulation efficiency;

*LC*: loading capacity;

*P_T_*: total PP content;

*P_S_*: PP content in supernatant;

*M*: mass of NPs-PP loaded.

### 3.5. Characterization of NPs-PP Loaded

#### 3.5.1. SEM Tracking

The morphology of the NPs-PP loaded was obtained by SEM [27]. The NPs-PP loaded was washed twice with distilled water and ethanol under centrifuge at 8000 rpm for 5 min, then resuspended in ethanol. The suspension was dropped onto conductive tape. After ethanol volatilization, the conductive tape was subjected to gold spray treatment, then observed with a scanning electron microscope (XL30, Philips, Amsterdam, The Netherlands) and photographed.

#### 3.5.2. DLS Tracking

The particle size distribution, polydispersity index and zeta potential were determined using a ZetaPALS Zeta Potential Analyzer (BIC BROOKHAVEN INSTRUMENTS CORPORATION, New York, NY, USA) at room temperature following the method by Stetefeld [28]. Each measurement was performed in triplicate.

#### 3.5.3. FT-IR Spectroscopy

FT-IR spectra (Bruker IFS55FTIR, Ettlingen, Germany) were recorded using the KBr method to identify the functional groups that might be involved in the formation of the nanoparticles to investigate the interaction between the two polyelectrolytes [19]. The samples (2 mg) and KBr (140 mg) were mixed and ground, and then, the spectrum range of 4000–400 cm^−1^ was taken by FT-IR spectrometer.

### 3.6. In Vitro Release Kinetics Profile

The NPs-PP loaded release kinetics profile was conducted with slight modification of Li’s method [29]. Briefly, the samples (200 mg) were placed in SGF (2 g/L sodium chloride, 3.2 g/L pepsin, pH 1.2) or SIF (6 g/L potassium dihydrogen phosphate, 10 g/L pancreatin, pH 7.0) at 37 °C, respectively. The samples should be placed in SGF for 2 h before being placed in SIF. At proper time intervals, precipitations were removed by centrifugation (8000 rpm for 5 min, 4 °C) and the supernatants were analyzed spectrophotometrically by the modified Folin-Ciocalteu method for the determination of PP as described above. The equation was as follows: release rate (%) = (released PP/PP encapsulated) × 100.

To describe the release kinetics of PP from formulations, zero-order, first-order, Higuchi, Hixcon-Crowell and Ritger-Peppas mathematical models were used. The Ritger-Peppas model can be applied to elucidate the release profiles of drug delivery systems with different mechanisms and geometries, which was calculated using the following equation [29,30]:

*M_t_*/*M_i_* = *kt^n^*

where *M_t_*/*M_i_* is the fractional solute release at time *t*, *k* is the rate constant and *n* is the release exponent characteristic of the release mechanism. The limitation of the exponent *n* value is 0.43 under pure Fickian release. The *n* value is between 0.43 to 0.85 when non-Fickian (anomalous) diffusion dominates. The adequacy of the delivery profiles for the mathematical models is based on the value of correlation coefficient (R^2^).

### 3.7. Simulated Microgravity

A rat hindlimb unloading model was used to simulate the microgravity effect in vivo [31]. Male Sprague-Dawley rats (200 ± 20 g, 6 weeks) were obtained. All animal experiments comply with the National Institutes of Health guide for the care and use of laboratory animals (NIH Publications No. 8023, revised 1978). The protocol was approved by the ethical committee of the Harbin Institute of Technology Laboratory Animal Center. The approval number of the ethics committee that endorsed this study is IACUC-2021045. A total of 50 rats were randomly divided into five groups (CK, HU, NP, NPs-PP loaded and PP, 10 rats for each group) after 1 week of acclimatization to living conditions. Hindlimb unloading was performed in each group except CK. CK and HU were gavaged with distilled water, while NPs were gavaged with NPs without PP loaded at a dose of 118.75 mg/kg/d (equivalent to NPs-PP loaded at 156.25 mg/kg/d), NPs-PP loaded at a dose of 156.25 mg/kg/d (equivalent to PP 37.5 mg/kg/d), and PP at a dose of 37.5 mg/kg/d, respectively, since the first day that hindlimb-unloading was performed. After 30 days, bilateral femurs, serums and colon tissues were harvested after all rats were sacrificed. Femurs were fixed with 75% alcohol at −20 °C. Serums were stored at −80 °C.

### 3.8. Hematoxylin and Eosin (HE) Staining

Liu’s method was used to observe colon tissue morphology [32]. Harvested colon tissues were fixed in 4% paraformaldehyde. The tissues were dehydrated and embedded in paraffin. After the paraffin block, fixed tissues were sliced into sections of 5 μm thickness and were mounted on glass slides, deparaffinized in xylene and rehydrated through graded alcohols, before staining with HE for the histological examination. Image J software was applied for detecting indicators of intestinal morphology and structure.

### 3.9. Ex Vivo Mucosal Adhesion Studies of NPs-PP Loaded 

NPs-PP loaded adhesive property detection was conducted according to Yan’s method [33]. Colon tissues were washed with normal saline and cut into segments of 3 cm in length immediately after rats were sacrificed. Segments were opened longitudinally along the mesentery and incubated with 1 mL of NPs-PP loaded for 2 h at 37 °C. The adhesion rate is calculated by the number of microspheres shed under the microscope:

Adhesion (%) = (Number of microspheres added − Number of microspheres shed)/Number of microspheres added ∗ 100%

### 3.10. Micro-CT Detection

The micro-CT (viva CT 40, Scanco Medical, Zurich, Switzerland) was used to analyze the microscopic structure of cancellous bone in the distal femur. Briefly, the femur was vertically aligned with the scan axis. The spatial resolution of the sample scan was set to 5 µm per voxel. The femur was identified with a semi-automatically drawn contour at each two-dimensional (2D) section. A distal femoral growth plate and above 2.1 mm of cancellous area were set as regions of interest (ROI) and 200 serial slices were used to establish a 3D reconstructed image to quantitatively calculate the microstructural morphological parameters of distal cancellous bone through the image analysis program of the µ-CT workstation.

### 3.11. BALP and PINP Analysis

Concentrations of BALP and PINP in serum samples were determined using an enzyme-linked immunoassay detection kit (SHANGHAI SUER BIOLOGICAL TECHNOLOGY CO., LTD, Shanghai, China).

### 3.12. Statistical Analysis

All data in this study were statistically analyzed by ORIGIN 9.0 software. The results were expressed as mean ± standard deviation. One-way ANOVA was used to compare differences among groups. *p* values < 0.05 were considered statistically significant. The data in this study were obtained by three independent experiments.

## 4. Discussion

During the space mission, physiological changes in intestinal mucosa in astronauts may lead to the decrease in or complete loss of the efficacy of many protective agents [34]. There is limited research on astronaut food additives which serve as gastrointestinal deliver systems under microgravity. In this study, *Auricularia auricula* anionic polysaccharide was used to construct nanoparticles with a rough surface to serve as food additives for PP delivery through the gastrointestinal tract. Such research is rarely reported in this field.

In this paper, the *Auricularia auricula* polysaccharide fragments carry negative charge after exchange with NaCl, and they can serve as an anionic polymer to form PECs with a cationic polymer. Because the literature has shown that PECs with a rough surface have a better mucosa adhesion [35], formulations of NPs-PP loaded were studied first to construct the rough structure on the nanoparticles. From this angle, the method for PEC construction that is commonly used was abandoned. And the SEM tracking method was used to define the concentration range of AAP Iα, ε-PL and PP. The data showed that the three components had different contributions to the rough surface of PEC construction. ε-PL had the narrowest concentration range, so we speculated that ε-PL was the main factor in forming nanoparticles. In addition, due to the widest concentration range of AAP Iα, and the fact that AAP Iα had the highest content in the surface structure of NPs-PP loaded which was exhibited in the FT-IR spectrum, we inferred that AAP Iα had prominent contributions to the surface construction. There was no significant effect of PP on NPs-PP loaded formation and surface structure construction.

The properties of dispersibility, stability and loading contents are key parameters for carrier practical application. Therefore, the size distribution, PDI and zeta potential of NPs-PP loaded was studied first. In a comprehensive comparison, lower PDI and zeta potential was exhibited in NPs-PP loaded with a narrower size distribution. The opposite situation was observed in NPs-PP loaded with a wider size distribution. This trend is consistent with the findings of Zheng’s research [36]. However, it was found that the assembly mechanism could be changed following concentration manipulation in AAP Iα and ε-PL, and different NPs-PP loaded structures were formed subsequently. Both low AAP Iα and ε-PL concentrations and high ε-PL concentrations resulted in the appearance of two different sized nanoparticle clusters. Because AAP Iα mainly comes from polysaccharide fragments washed out by low-concentration NaCl in ion exchange chromatography, AAP Iα carries a weak negative charge. It could be deduced that AAP Iα belongs to polyelectrolytes with a low charge density, while ε-PL belongs to a high charge density type of polyelectrolyte. Different assembly forms can be achieved when a similar molecular weight appeared between polyelectrolytes with low and high charge density [37]. In the present study, the molecular weight between AAP Iα and ε-PL was very similar, and there were significant changes in size distribution, PDI and zeta potential with ε-PL concentration increases, indicating that changes emerged in the PEC assembly mechanism. Furthermore, with increasing AAP Iα concentration, the zeta potential of NPs-PP loaded slowly decreased. Based on these, we speculate that the influence of ε-PL on the assembly mechanism was particularly prominent. Meanwhile, the trends of AAP Iα, ε-PL and PP concentrations on loading content of the NPs-PP loaded were consistent with the data of size distribution, PDI and zeta potential; however, the three had a weak effect on the loading content of the nanoparticles (21.87 ± 0.12% to 23.19 ± 0.12%). In addition, Phenolic hydroxyl groups which appeared in PP can form hydrogen bonds with the hydroxyl groups in AAP Iα and the carbonyl groups in ε-PL, respectively. So, the phenomenon of little change in loading content may suggest that the assembly process may be divided into two steps. For one step, PP may first form electrostatic interactions with AAP Iα and ε-PL, and AAP Iα-PP and ε-PL-PP are assembled, respectively. In step two, PECs are assembled by AAP Iα-PP and ε-PL-PP.

In the FT-IR spectrum, AAP Iα was first identified as having a polysaccharide structure because a wide C-OH absorption peak distributed from 3000 to 3600 cm^−1^ was observed [38]. In addition, the sharp peak at 1622 cm^−1^ represented the absorption of C=O [39]. The C-O of the carboxyl group appeared around 1420 cm^−1^ with a weak peak [40]. These results proved that AAP Iα was a polysaccharide structure with -COOH. The characteristic peaks of polyphenol compounds disappeared on the NPs-PP loaded FT-IR spectrum, indicating that PP was encapsulated in PECs. And the phenomenon of a new absorption peak at 1602 and 1563 cm^−1^ on the NPs and NPs-PP loaded spectra, respectively, indicates that electrostatic interactions are the intermolecular forces between AAP Iα and ε-PL [41]. All of these data demonstrate that PP was encapsulated in PECs which was formed by AAP Iα and ε-PL through electrostatic interactions, while AAP Iα was the main component of surface structure on NPs-PP loaded.

**Figure 9:**
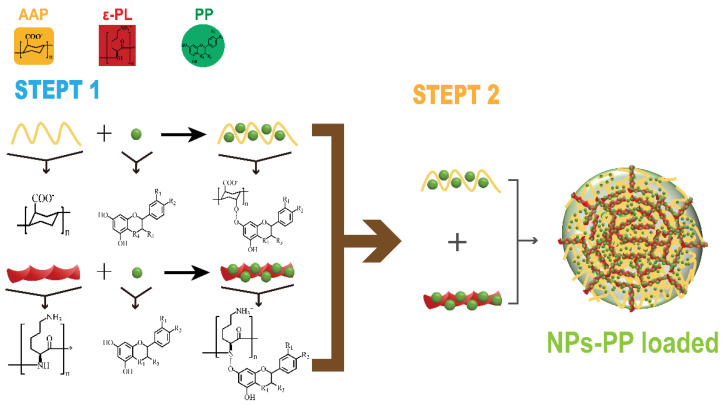
Mechanism of NPs-PP loaded formation.

To further investigate the assembly mechanism of NPs-PP loaded, the PP release profiles in SGF and SIF were studied. Based on the Peppas equation ($\frac{M_{t}}{M_{i}}={kt}^{n}$) [42,43], the dissolution mechanism of NPs-PP loaded was discussed. The above equation was transformed into the Ritger-Peppas equation, and the n value was equivalent to the slope of the Ritger-Peppas equation. The data showed that NPs-PP loaded exhibited non-Fickian diffusion, indicating that the dissolution pattern was the result of the synergistic effect of diffusion and skeleton erosion. Combining the results of SEM and DLS, we speculated that ε-PL acted as the skeleton system of NPs-PP loaded, and AAP Iα filled the inside of the ε-PL skeleton system. Because loose PECs often form rough surface structures [37], we speculated that AAP Iα and ε-PL form nanoparticles with loose structures. The absorption peak at 1563 cm^−1^ in the FT-IR indicates the occurrence of electrostatic interactions between AAP Iα and ε-PL in the nanoparticle surface structure. Therefore, we inferred that ε-PL extends as the skeleton system to the surface of PECs. Based on the above conclusions, we confirmed that the assembly process of NPs-PP loaded was completed in two steps. First, PP forms hydrogen bonds with the hydroxyl groups in AAP Iα and the carbonyl groups in ε-PL, respectively. Second, AAP Iα and ε-PL, which carry PP molecules, respectively, form a loose PECs with a skeleton system of ε-PL through complex electrostatic interactions (Figure 9).

Most polyphenolic compounds are absorbed in the colon [44]. Moreover, the tissue structure of the colon has a significant impact on the adhesion of nanoparticles [35]. This paper found that under simulated weightlessness, a decrease in the depth of colonic crypt resulted in a decrease in surface roughness and surface area of colonic tissue. Reduced surface roughness in colon tissue leads to reduced adhesion of nanoparticles to the surface of colon tissue, because PECs with polysaccharides as the surface can exhibit good mucosal adhesion characteristics [45]. Therefore, from a structural point of view, the surface of the nanoparticles constructed in this study is AAP I α, which can increase the adhesion of nanoparticles to colonic mucosa. Although the results suggest that the adhesion of NPs-PP loaded to the colon decreases under simulated weightlessness, considering that the surface area of the colon decreases with simulated weightlessness, therefore, when the surface area of the colon was represented by crypt depth, there was no significant difference between the ground and simulated weightlessness groups. The results of the adhesion properties revealed that adhesion ability of NPs-PP loaded was unchanged despite the colon structural change.

To validate the delivery effect of NPs-PP loaded under simulated microgravity, the mechanical properties of femurs were first tested. Although the results showed no significant difference in the protective effect of NPs-PP loaded and PP on the mechanical properties, we noticed that the mechanical properties in the NPs-PP loaded group were superior to those in the PP group. The mechanical properties were greatly affected by the microstructure of the skeleton system [46], so micro-CT was used to study the microstructure of the femur. The results showed that in both the degree of order in the CADF and the quantitative analysis after 3D reconstruction, the protective effect of NPs-PP loaded on the CADF was significantly better than that of PP. The results of BALP and PINP further proved that PECs which were assembled by AAP Iα and ε-PL could significantly improve the bioavailability of PP.

## 5. Conclusions

In conclusion, the literature on food additives as gastrointestinal delivery systems in astronaut food is very rare. In the present study, AAP Iα as an anionic polysaccharide fragment was isolated from *Auricularia auricula* through an encapsulation efficiency tracking method. One novel food additive which was presented as PECs with a negative charge was assembled by AAP Iα and ε-PL. And the PECs served as carriers for PP gastrointestinal delivery under microgravity. NPs-PP loaded were formed with a rough surface after PP encapsulation. AAP I α can interact strongly with ε-PL to form stable and homogeneous nanoparticles. NPs-PP loaded with a rough surface and an ε-PL skeleton structure gave loose PECs formed by electrostatic interactions between AAP I α-PP and ε-PL-PP. Additionally, the rough surface formed by AAP Iα enabled PECs to have good colon adhesion properties and enhanced PP bioavailability. These findings proved that AAP Iα-ε-PL PECs could encapsulate, stabilize and deliver PP effectively under microgravity. This work could contribute to the research and development of aerospace and functional foods.

## Figures and Tables

**Table 1:** Analysis of GPC.

Items	Results
Mn (Daltons)	110,144
Mw (Daltons)	133,304
Mz (Daltons)	151,340
Mp (Daltons)	145,663
Mw/Mn	1.210
Peak RV	9.211

**Table 2:** PDI of NPs-PP loaded.

	Concentration (μg/mL)	PDI
AAP Iα	400	0.232 ± 0.011
	500	0.197 ± 0.015
	600	0.193 ± 0.008
	700	0.157 ± 0.01
	800	0.151 ± 0.009
	900	0.176 ± 0.007
ε-PL	10	0.235 ± 0.013
	15	0.213 ± 0.015
	20	0.157 ± 0.008
	25	0.165 ± 0.012
	30	0.211 ± 0.009
	35	0.225 ± 0.01
PP	40	0.164 ± 0.008
	50	0.162 ± 0.011
	60	0.158 ± 0.009
	70	0.163 ± 0.01
	80	0.16 ± 0.012
	90	0.168 ± 0.007

**Table 3:** Release kinetic models of loaded NP-PP.

Formulation	Kinetic Model	Regression Equation	Slope	R^2^
NP-PP loaded (SGF)	Zero-order	M_t_/M_i_ = 0.0066t + 0.0295	0.0066	0.904
	First-order	Ln (1 − M_t_/M_i_) = −0.007t − 0.0298	−0.007	0.9063
	Higuchi	M_t_/M_i_ = 0.0237t^1/2^ + 0.0098	0.0237	0.9613
	Hixcon-Crowell	(1 − M_t_/M_i_)^1/3^ = −0.0023t + 0.9901	−0.0023	0.9056
	Ritger-Peppas	Ln (M_t_/M_i_) = 0.4189Ln t − 3.4341	0.4189	0.9777
NP-PP loaded (SIF)	Zero-order	M_t_/M_i_ = 0.1036t + 0.2427	0.1036	0.9792
	First-order	Ln (1 − M_t_/M_i_) = −0.3111t + 0.0264	−0.3111	0.9083
	Higuchi	M_t_/M_i_ = 0.917t^1/2^ − 0.6413	0.917	0.9583
	Hixcon-Crowell	(1 − M_t_/M_i_)^1/3^ = −0.0703t + 0.9595	−0.0703	0.9477
	Ritger-Peppas	Ln (M_t_/M_i_) = 0.5277Ln t − 1.1293	0.5277	0.9822

**Table 4:** Formulations of NPs-PP loaded.

Formulation	AAP Iα (μg/mL)	ε-PL (μg/mL)	PP (μg/mL)
A	400	20	90
B	500	20	90
C	600	20	90
D	700	20	90
E	800	20	90
F	900	20	90
G	800	10	90
H	800	15	90
I	800	20	90
G	800	25	90
K	800	30	90
L	800	35	90
M	800	20	40
N	800	20	50
O	800	20	60
P	800	20	70
Q	800	20	80
R	800	20	90

## Data Availability

Data will be made available on request.

## Supplementary Materials

The following supporting information can be downloaded at: https://www.mdpi.com/article/10.3390/molecules29010245/s1, Figure S1: The spectrum of GPC on AAP Iα, Figure S2: Effect of NPs-PP loaded on mechanical properties of rat femur.

## Abbreviations

AAP	Crude polysaccharides of *Auricularia auricular*
AAP Iα	an anionic polysaccharide fragment isolated from *Auricularia auricula*
ε-PL	ε-poly-L-lysine
PP	*Pinus koraiensis* polyphenol
PECs	polyelectrolyte complexes
NPs-PP loaded	nanoparticles formed by AAP Iα and ε-PL loaded with PP
SEM	scanning electron microscope
DLS	dynamic light scattering
FT-IR	Fourier-transform infrared
PDI	Polydispersity index
SGF	simulated gastric fluid
SIF	simulated intestinal fluid
CK	Ground control
HU	Hindlimb unloading
CADF	cancellous area from distal femur
BALP	bone alkaline phosphatase
PINP	procollagen type I N propeptide
