# Supplementary material for: Auricularia auricula Anionic Polysaccharide Nanoparticles for Gastrointestinal Delivery of Pinus koraiensis Polyphenol Used in Bone Protection under Weightlessness
Source: Molecules. 2024 Jan 2;29(1):245. doi: 10.3390/molecules29010245 (PMC10780251; doi:10.3390/molecules29010245)
Supplement: Supplementary file 1 [file molecules-29-00245-s001.zip › molecules-2765312-supplementary.pdf]

Supplementary Materials

Auricularia auricula Anionic Polysaccharide Nanoparticles for Gastrointestinal Delivery of Pinus koraiensis Polyphenol Used in Bone Protection under Weightlessness

Supplementary Figures

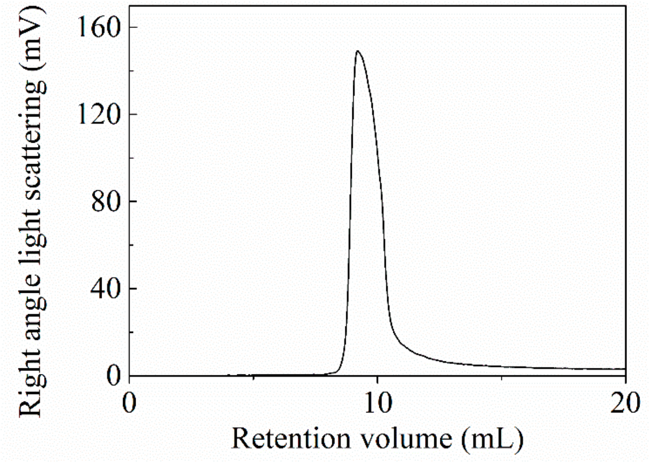

Figure S1. The spectrum of GPC on AAP Iα

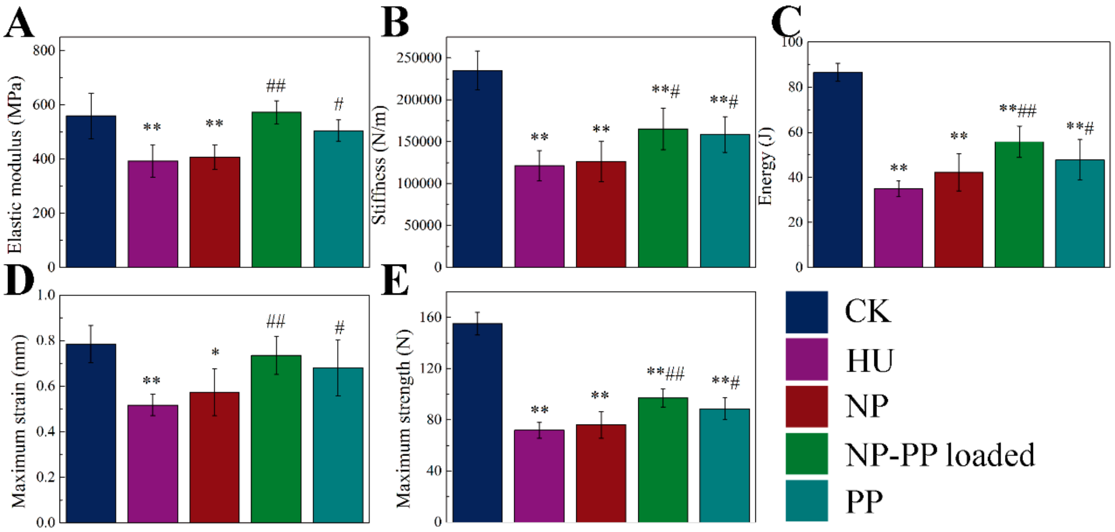

Figure S2. Effect of NPs-PP loaded on mechanical properties of rat femur. The statistical results shown represent the mean ± SD; vs. the control group *$P < 0.05$, **$P < 0.01$; vs. the HU group #$P < 0.05$, ##$P < 0.01$; vs. the PP group △$P < 0.05$, △△$P < 0.01$; n = 6 rats for each group. CK: ground group; HU: hindlimb-unloaded group; NP: rats were treated with 118.75 mg/kg/d nanoparticles without PP loaded during hindlimb- unloaded; NP-PP loaded: rats were treated with 156.25 mg/kg/d nanoparticles with PP loaded during hindlimb-unloaded; PP: rats were treated with 37.5 mg/kg/d polyphenols isolated from pine (*Pinus koraiensis*) during hindlimb- unloaded.
